# Same same but different—Image‐based versus imageless robotic‐assisted total knee arthroplasty!

**DOI:** 10.1002/jeo2.70062

**Published:** 2024-10-19

**Authors:** Michael T. Hirschmann, George Avram, Heiko Graichen, Reha N. Tandogan, Natalie Mengis, Stefano Zaffagnini

**Affiliations:** ^1^ Department of Orthopaedic Surgery and Traumatology Kantonsspital Baselland Bruderholz Switzerland; ^2^ Department of Clinical Research Research Group Michael T. Hirschmann, Regenerative Medicine & Biomechanics, University of Basel Basel Switzerland; ^3^ Department of Personalised Orthopaedics (PersO) at Privatklinik Siloah Berne Switzerland; ^4^ Department of Orthopaedics and Traumatology Çankaya Hospital Ankara Turkey; ^5^ Department of Orthopaedics and Traumatology Halic University Istanbul Turkey; ^6^ Department of Orthopaedic Surgery and Traumatology Clinica Ortopedica e Traumatologica II, IRCCS Istituto Ortopedico Rizzoli, c/o Lab Biomeccanica ed Innovazione Tecnologica Bologna Italy; ^7^ DIBINEM, University of Bologna Bologna Italy

**Abbreviations**
3D: three dimensional
ACL: anterior cruciate ligament
CT: computed tomography
CTKA: conventional total knee arthroplasty
PROMs: patient‐reported outcome measures
ROM: range of motion
RTKA: robotic‐assisted total knee arthroplasty
TKA: total knee arthroplasty

## INTRODUCTION

Robotic‐assisted total knee arthroplasty (RTKA) has gradually become a widely available procedure due to the increasing evidence that it provides better accuracy compared to conventional techniques (conventional total knee arthroplasty) [4, 24, 29, 30]. While the literature has yet to provide decisive evidence on the influence of robotic assistance on postoperative patient‐reported outcome measures (PROMS) following TKA [2, 14, 17], the expected accuracy in all three planes has the potential to lead to improved postoperative joint kinematics [4, 13, 30] and range of motion (ROM) [12], thereby providing an overall higher patient function and satisfaction [14]. Nonetheless, controversy still exists on whether there is a link between robotic accuracy and joint kinematics and postoperative outcomes. While many surgeons doubt that accuracy per se is the key to a forgotten joint, alignment approaches seem to make the difference, especially in terms of soft‐tissue balancing [8, 26]. For this reason, accuracy as a main target feels misleading when, in fact, it is only a prerequisite for successfully delivering advanced alignment solutions. Today, the thought that accuracy alone is the main focus of robotic surgery seems to be proven wrong by the body of knowledge provided by years of dedicated research into navigation technology [15, 16].

Since the advent of robotics in orthopaedic surgery, two main robotic systems can be differentiated: image‐based [30] and image‐less RTKA [4].

Image‐based RTKA uses peri‐articular motion‐capture optical trackers and intraoperative bone mapping to align bony landmarks with a preoperative computed tomography (CT) scan. By matching the digitized bony references with a CT scan, the surgeon gets real anatomical information about the patient's knee, and the robotic system allows accurate planning and TKA implantation [6]. In contrast, image‐less RTKA systems rely solely on intra‐articular point clouds digitized from the bony knee and ankle as well as a calculation of the hip centre. This data is then matched to multiple knee models in a best‐fit manner and virtually displayed to the surgeon [25].

Both additionally assess the joint gaps allowing an appreciation of knee joint laxity.

Table 1 presents the most common landmarks recorded intraoperatively during robotic‐assisted surgery.

**Table 1:** Common reference points registered intraoperatively during RTKA.

**Image‐less RTKA** a	**Image‐based RTKA** b
**Femur**	
Medial and lateral condyles—point cloud using the most distal point on each condyle as a reference	A combination of anterior‐distal and posterior point clouds, as recommended by the robotic system
Medial and lateral condyles—point cloud using the most posterior point on each condyle as reference	
Anterior cortical surface	
Knee centre	*Internal computation of the knee centre*
Medial and lateral epicondyles	*Determined preoperatively during the planning process*
Whiteside's line	‐

*Tibia*	
Medial and lateral malleoli	Medial and lateral malleoli
Medial and lateral plateau points	A point‐cloud mask generated by the summation of medial and lateral plateau, anterior/tibial tuberosity and deep antero‐medial tibial plateau registration points.
Tibia centre—ACL footprint	*Internal computation of the tibia centre*
Sagittal axis	*Determined preoperatively during the planning process*
Both systems currently have no information on the patella's surface or its orientation in relation to the trochlea.	

Abbreviations: 3D, three dimensional; ACL, anterior cruciate ligament; CT, computed tomography; RTKA, robotic‐assisted total knee arthroplasty.

^a^ Image‐less RTKA uses point‐cloud registration to generate a standardized template of the patients' intra‐articular surface, a process called morphing [25].

^b^ Image‐based RTKA uses a specific, fixed number of intraoperatively recorded points directly match the knees' anatomy to the 3D CT recorded preoperatively.

When it comes to comparing the two systems, the literature has yet to provide conclusive evidence in terms of accuracy, PROMs, ROM and postoperative patient satisfaction [27]. Specifically, when accuracy is concerned, it seems that there are no significant differences between the two systems in terms of femoral and tibial coronal plane alignment [9, 20, 21, 22, 28, 31]. Nonetheless, there seem to be differences between planned and achieved bone cuts between the two systems. Specifically, the femoral and tibial coronal cuts seem to be more prone to be unintentionally in varus for image‐less systems compared to image‐based [28]. This might be due to the fact that image‐less systems rely on intra‐articular anatomy for reference, and because of this, placing the right intra‐articular landmarks is paramount for accurate implantation; otherwise, the system registers a different joint line compared to the native one. In both techniques, it is important to understand the location of the worn cartilage and the extent of cartilage wear because both aim to restore the joint surface as closely as possible, although they do so in different ways. Image‐based systems work with real three‐dimensional (3D) patient models, while image‐less systems adapt generic 3D models from the software to match the intra‐articular anatomy and generate reference planes. As a result, image‐based systems can detect actual resection thickness, whereas image‐less systems focus on accurately orienting cutting angles within predefined planes. Namely, when dealing with a varus knee, the medial cartilage surfaces are worn, and therefore, the joint surface reference starts off more into varus than it normally would. In the sagittal plane, specifically regarding the reconstruction of the native tibial slope, image‐free systems face certain limitations. This is because they only capture a few points intraoperatively. While these points suffice for accurate implantation within the defined planes, these planes do not always correspond to the patient's native knee anatomy. As a result, image‐free systems generate 3D planes within which they accurately target specific angles. However, the actual thickness of each bone cut depends heavily on intraoperative mapping, particularly for the tibia, and less so for the distal femur. This limitation poses a challenge for kinematic or inverse kinematic alignment when using image‐less robotic systems, as these alignment methods rely on precise bone cut thicknesses. In contrast, patient‐specific or functional alignment focuses more on angles to guide implant orientation, potentially offering better results in these cases. On the other hand, image‐based systems rely on registering a larger number of points on the tibial surface, which facilitates the matching process between the preoperative 3D‐CT and intraoperative anatomy. This aspect might allow image‐based systems more leeway when dealing with alignment concepts that require precise bone cuts. At the same time, it is important to note that measurement errors reported using postoperative radiographs for both systems generally fall within the 1–2° ± 1.5° normal error range [9, 20, 21, 22, 28, 31]. This provides a useful perspective when assessing the accuracy of current robotic systems, as their reported errors generally fall within these same limits.

Assessing accuracy in current robotic systems has become increasingly complex due to the use of different alignment strategies, making it challenging to compare the accuracy of various systems when surgeons aim for different alignment targets. Additionally, most RTKA studies do not incorporate functional knee phenotypes into their methodology [11]. As a result, preoperative measurements, planned bone cut thicknesses, and angles are often pooled and compared to postoperative results, offering limited insight into each system's ability to reconstruct patient‐specific anatomy or actual surgical intent [10]—an indirect but important measure of surgical accuracy.

## THE FUTURE OF RTKA

Increasing evidence suggests that the knee's flexion‐extension facet and anterior compartments operate independently, without any significant correlation observed between compartments [5]. For this reason, the alterations to the knee joint surface imposed by current TKA implantation strategies often significantly alter prearthritic knee kinematics, particularly when using off‐the‐shelf implants [3]. Furthermore, achieving balanced and stable gaps is often a moving target [8] because different alignment strategies and their corresponding bone cuts must accommodate standardized implants. These implants frequently struggle to adapt to each patient's specific anatomy [7] often resulting in asymmetric ligament imbalances, which, if not properly addressed, can result in residual discomfort or pain after TKA [19].

By considering the changes taking place in the osteoarthritic knee in terms of articular surface change, RTKA might offer new insights into developing different implantation strategies [23] that are optimized for widely available off‐the‐shelf implants. By identifying implant positioning solutions that minimize postoperative knee joint surface changes compared to the preoperative state [18], surgeons can move beyond classical alignment parameters, which are challenging to manage accurately using conventional methods [1, 27].

## CONFLICT OF INTEREST STATEMENT

The authors declare no conflict of interest.

## ETHICS STATEMENT

The authors have nothing to report.
